# Editorial: Heparin and Related Polysaccharides

**DOI:** 10.3389/fmed.2020.00211

**Published:** 2020-05-22

**Authors:** Elaine Gray, David A. Keire, Barbara Mulloy, Timothy R. Rudd

**Affiliations:** ^1^National Institute for Biological Standards and Control, Potters Bar, United Kingdom; ^2^Institute of Pharmaceutical Science, King's College London, London, United Kingdom; ^3^Division of Pharmaceutical Analysis, Office of Testing and Research, Center for Drug Evaluation and Research, United States Food and Drug Administration, St Louis, MO, United States

**Keywords:** bovine heparin, porcine heparin, analysis, NMR, chromatography, recombinant heparin, thrombin

The 7th Workshop on the Characterization of Heparin Products was held in London in December 2017, organized by the UK's National Institute for Biological Standards and Control (NIBSC) and the United States Pharmacopeial Convention (USP) and sponsored by King's College London. This heparin meeting was the latest in an occasional series of workshops designed to bring together participants representing heparin manufacturers, regulatory bodies, and methods development analysts in this field to discuss current topics and future strategies for heparin production.

Heparin is a widely used lifesaving anticoagulant drug of biological origin. Unlike many other drugs heparin is not a small molecule, peptide, or protein but a linear sulfated polysaccharide categorized as a glycosaminoglycan. In addition to being used as a clinical medicine, heparin with its complex mixture of polyanionic glycosaminoglycan chains is the subject of much academic study. A heparin contamination crisis in 2007-2008 (1) resulted in many fatalities, and as a consequence, physicians, heparin manufacturers, regulatory bodies, and academic researchers all contributed to an effort to modernize the quality control analysis of heparin in clinical use (2). The Heparin Workshop series represents a continuation of the close collaboration of subject matter experts from many backgrounds that began in 2007.

The 7th Workshop was divided into sessions including those on heparin characterization, bovine heparin, regulatory perspectives, and the future of heparin and the papers in this special Research Topic collection reflect those subjects. The keynote presentation, given by Hemker et al. of Maastricht University in the Netherlands proposed a radically new approach to the measurement of anticoagulant activity of heparin based on the endogenous thrombin potential and the concentration of the active, thrombin inhibiting sequence in the heparin sample in question.

At present heparin is prepared from porcine intestinal mucosa, but the current prevalence of African Swine Fever, particularly in Asia, threatens this source. A potential alternative source is bovine intestinal mucosa, previously avoided on the grounds of potential prion contamination. Given a prion-free source of bovine material, the next step is to explore the spectrum of structural and functional properties of bovine mucosal heparin, which is not identical with the porcine product. Suitable compendial methods and specifications need to be drafted, and the story of how this might be accomplished is outlined by Vilanova et al. in their paper on the two monographs on porcine and bovine heparin respectively that are now established as part of the Brazilian Pharmacopeia. In addition, Workman and Carrick of the US Pharmacopeia present methods for the analysis of impurities in bovine heparin and compare results between porcine and bovine material. In terms of anticoagulant function, porcine heparin has a higher specific activity than bovine heparin, but when dosed in terms of units of activity the two preparations are equivalent in their capacity to prevent thrombosis (Jeske et al.).

Methods to distinguish between heparin samples originating from different species are in development, and one of these uses the powerful technique of NMR spectroscopy. In the study by Mauri et al. spectra of heparin from bovine, porcine and ovine intestinal mucosa and from bovine lung were compared; because of their unique tissue specific monosaccharide compositions, 1D or 2D NMR data can be used to clearly identify the species and organ source of each type of sample. The compositional analysis of heparins, in terms of its constituent disaccharides, can also be achieved using chromatographic techniques as well as by 2D heteronuclear NMR, and the two very different approaches were compared in the study of Spelta et al.. In some respects, the two techniques yield complementary information, and their particular advantages and drawbacks are discussed. The extraction and purification of heparin from its biological source involves high temperatures and high pH steps which can leave their mark in the fine structure of commercially prepared heparin, and the resulting process-related impurities can affect the quality of the final product. Anger et al. present a chromatographic method for the specific set of structures that have been partially desulfated, pointing out its utility for the improvement of quality control in heparin manufacture.

The final session of the Heparin Workshop looks to the future for solutions to the difficulties inherent in the biological source of heparin. The production of heparin-like compounds in CHO cells, as described by Glass, can be achieved at present to a good standard of structural consistency. Such novel compounds could in theory be engineered to specific functional targets, generating predictable biological activities and avoiding harmful side-effects. There is no longer any doubt that this is possible; manufacture of heparin-like products by synthetic, semi-synthetic, and cell-based methods is now only held back by the need to produce high quantities of material at a cost-effective price.

## Author Contributions

All authors listed have made a substantial, direct and intellectual contribution to the work, and approved it for publication.

## Conflict of Interest

The authors declare that the research was conducted in the absence of any commercial or financial relationships that could be construed as a potential conflict of interest.
